# Pandemic and student mental health: mental health symptoms among university students and young adults after the first cycle of lockdown in the UK

**DOI:** 10.1192/bjo.2022.523

**Published:** 2022-07-26

**Authors:** Nicole K. Y. Tang, Katharine A. M. McEnery, Laura Chandler, Carla Toro, Lukasz Walasek, Hannah Friend, Sai Gu, Swaran P. Singh, Caroline Meyer

**Affiliations:** Department of Psychology, University of Warwick, UK; Warwick Manufacturing Group, University of Warwick, UK; Wellbeing and Safeguarding Group, Professional Services, University of Warwick, UK; Executive Office and School of Engineering, University of Warwick, UK; Division of Mental Health and Wellbeing, Warwick Medical School, UK

**Keywords:** University students, young adults, mental health, well-being, COVID-19

## Abstract

**Background:**

Early COVID-19 research suggests a detrimental impact of the initial lockdown on young people's mental health.

**Aims:**

We investigated mental health among university students and young adults after the first UK lockdown and changes in symptoms over 6 months.

**Method:**

In total, 895 university students and 547 young adults not in higher education completed an online survey at T1 (July–September 2020). A subset of 201 university students also completed a 6 month follow-up survey at T2 (January–March 2021). Anxiety, depression, insomnia, substance misuse and suicide risk were assessed.

**Results:**

At T1, approximately 40%, 25% and 33% of the participants reported moderate to severe anxiety and depression and substance misuse risk, clinically significant insomnia and suicidal risk. In participants reassessed at T2, reductions were observed in anxiety and depression but not in insomnia, substance misuse or suicidality. Student and non-student participants reported similar levels of mental health symptoms. Student status was not a significant marker of mental health symptoms, except for lower substance misuse risk.

Cross-sectionally, greater symptoms across measures were consistently associated with younger age, pre-existing mental health conditions, being a carer, worse financial status, increased sleep irregularity and difficulty since lockdown. Longitudinally, T2 symptoms were consistently associated with worse financial status and increased difficulty sleeping at T1. However, these associations were attenuated when baseline mental health symptoms were adjusted for in the models.

**Conclusions:**

Mental health symptoms were prevalent in a large proportion of young people after the first UK lockdown. Risk factors identified may help characterise high-risk groups for enhanced support and inform interventions.

The impact of COVID-19 restrictions on university students’ mental health and well-being during the initial phase of the pandemic has been investigated internationally, with varying rates of clinical problems reported depending on the location, timing and methods of the study. For example, in Guangdong, China, Li et al^1^ surveyed 164 101 university students in February 2020. Using the Patient Health Questionnaire 9 (PHQ-9) and General Anxiety Disorder 7 (GAD-7), the authors reported the prevalence rates of probable clinical depression and anxiety to be 22.4% and 12.1%. Using the same instruments, higher rates of clinical depression (48.1%) and anxiety (38.5%) were reported by Wang et al^2^ in May when they surveyed 2031 students within a large university system in Texas, USA. In France, Wathelet et al^3^ surveyed 69 054 university students between April and May. Using the Beck Depression Inventory-II and the State-Trait Anxiety Inventory-State subscale, the prevalence rates of severe depression and anxiety were estimated to be 16.1% and 27.5%.^4^ In addition, based on a separate one-item measure, 11.4% of the student participants reported suicidal thoughts in the past month. The rate of suicidal ideation was comparable to the 12.8% prevalence reported in a Bangladeshi study that surveyed 3331 university students in the same months^4^ but higher than the 8.2–9.8% prevalence of suicidal thoughts reported in a UK panel study with 3077 adults.^5^ There are legitimate concerns that pandemics could intensify known triggers of suicide risk in vulnerable groups,^5,6^ resulting in an increase in death by suicide following viral disease outbreaks.^7^

These high rates of depression, anxiety, and suicidal thoughts are thought to be attributable to the lockdown experience and appear responsive to changes in restriction policies. In a UK cohort study, Savage et al^8^ measured university students’ mental well-being twice before (Oct 2019 and Jan 2020) and twice during (March and April 2020) the first UK lockdown. The authors observed a significant decline in self-reported levels of mental well-being, with females reporting lower levels. Evan et al^9^ found a similar decline in well-being, with over a third of students reporting clinically significant symptoms of depression during the lockdown (April–May 2020), compared with 15% before. A Polish study^10^ involving 7228 university students over five stages of assessment from March to April 2020 demonstrated a significant increase in depression as the pandemic progressed, particularly when the lockdown involved more pervasive social distancing, isolation and restriction of movement. Interestingly, an Italian study found that students’ reported levels of depression rapidly returned to pre-pandemic levels once restrictions were lifted.^11^

Aside from depression, anxiety and suicide risk, there were a number of early COVID studies examining self-reported changes in sleep patterns. In a meta-analysis of 44 studies from 13 countries, Jahrami et al^12^ found that the global pooled prevalence of sleep problems during that pandemic was 35.7% among the general population. Specifically among university students and staff, an Italian survey found no change in sleep duration, but there were general delays in bedtime and waking time, an increase in time taken to fall asleep, and a worsening of sleep quality and insomnia symptoms during the lockdown.^13^ These changes in sleep timing and quality were more pronounced in females, students and evening-type individuals. There were also unexpected findings from the USA about alcohol/substance use, with reports suggesting the lockdown was associated with an increase in drinking frequency but reductions in the quantity of alcohol consumption, heavy drinking and drunkenness.^14^ Favourable changes were also reported in a French survey of 3671 university students that found reductions in reported binge drinking and tobacco and cannabis use compared with before lockdown.^15^ However, few studies have examined these health behaviours alongside mental health outcomes or identified the common risk factors marking or predicting poor outcomes across the board.

The REsponding to COVID-19 by Enhancing Resilience in Students (RECOVERS) study was set up to investigate mental health among university students and young adults after the first UK lockdown and changes in symptoms over 6 months. It had two components: an online survey and a pilot intervention study. The current study focused on the findings of the survey, which aimed to identify and evaluate characteristics and behaviours that increased the risk of mental health symptoms as the pandemic persisted.

## Method

### Study design

**Fig. 1:**
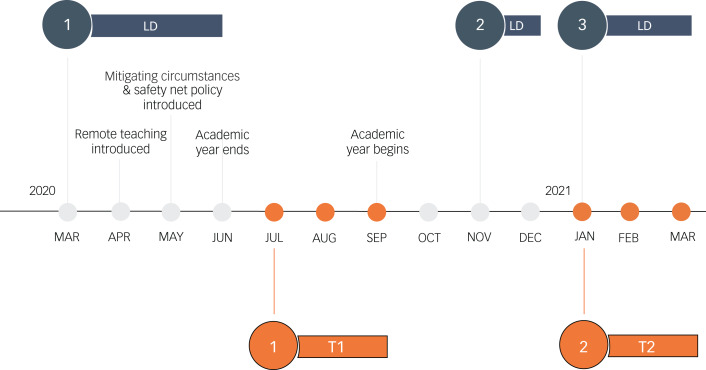
Timeline of COVID-19 and the Warwick RECOVERS study. Timeline of main events in the UK during the COVID-19 pandemic (lockdowns (LD) 1, 2 and 3), university conditions and time points of RECOVERS data collection. Mitigating circumstances and the safety net policy were university policies put in place to prevent students from being disadvantaged by the alternative teaching and assessment practices that arose from the COVID-19 pandemic. For example, an automatic 2 week deadline extension for assessments in progress (initiated before 13 March 2020) was introduced, and examination boards could be informed of how personal circumstances may have affected the production of research projects and take these circumstances into account when grading work.

The RECOVERS survey comprised two waves of data collection (Fig. 1). The first occurred between July and September 2020, just after the first UK lockdown and the final university term in the academic year. Unlike studies reported in the existing literature, the RECOVERS survey captured the experiences of participants during this later period of change and uncertainty as they navigated through the fluctuating restriction rules to return to some kind of ‘normality’. To explore the longer-term impact of the pandemic on mental health, a second wave of data collection was carried out between January and March 2021. A subgroup of students were surveyed during the third UK lockdown, during the academic spring term, as the vaccination programme was introduced.

Five mental health outcomes were investigated simultaneously at both time points to establish a broader profile of mental health: anxiety, depression, insomnia, substance misuse and suicidality. The baseline survey also examined contextual factors including demographic characteristics, pre-existing health status, study/work characteristics, risk and exposure to COVID-19, as well as changes in behaviours and circumstances during the pandemic. Changes included adjustments in study time, work hours, financial status, media engagement and sleep behaviours.

The RECOVERS survey examined the prevalence and risk factors of mental health symptoms cross-sectionally and longitudinally, using online questionnaires. The study protocol was informed by the research framework set out by Holmes et al^16^ and was developed with inputs from university student representatives and the university's well-being service. The authors assert that all procedures contributing to this work comply with the ethical standards of the relevant national and institutional committees on human experimentation and with the Helsinki Declaration of 1975, as revised in 2008. All procedures involving human subjects/patients were approved by the Humanities and Social Sciences Research Ethics Committee of the University of Warwick, UK. All participants provided informed consent prior to starting the online survey. It was made clear in the participant information sheet and at the start of the survey that participants were not obliged to complete all questions. They could close the browser any time, skip any questions or simply choose the ‘prefer not to say’ option. The participants were not given feedback on their questionnaire scores, as these measures were not designed to give diagnosis. However, a box containing links to helpline information appeared on each page of the questionnaire to ensure those who wanted to seek help could access the relevant services in a timely manner.

The cross-sectional component involved 895 university students (365 from a single university and 530 from other universities) and 547 young adults (aged 18–30 years) not in higher education. All participants completed a survey between July and September 2020 (T1).

The longitudinal component involved a subset of the university student participants recruited from a single university (*n* = 201) completing a 6 month follow-up survey between January and March 2021 (T2).

### Participants

Participants were recruited through two routes. Convenience sampling was used for participant recruitment at a single university, where the study was advertised through university channels. These student participants were not paid to take part in the study, but a prize draw of four £100 Amazon vouchers was introduced in August 2020 to incentivise participation. Prolific – an online participants recruitment platform – was used to recruit participants from other UK universities and non-student young adults living in the UK. Participants recruited through Prolific were paid the recommended rate (£6.50/h) to complete the T1 survey.

The inclusion criteria for university student participants were to be at least 18 years old and enrolled at a higher education institution in the UK. Non-student participants were required to be between 18 and 30 but not enrolled in full-time university education, forming a broadly ‘age-matched’ comparison as 92% of our university student participants fell in this age bracket. The upper age limit of 30 also matched well with the UK public perception of the boundaries of youth.^17^

A subgroup of the student participants who completed the T2 survey were recontacted because they had provided consent to do so. This subsample was drawn from those students recruited from a single university in the UK.

### Measures

The T1 and T2 surveys were identical, except that the T1 survey also asked participants about their demographics, study/work characteristics and pre-existing health status, and included questions on their risk and exposure to COVID-19 and changes in behaviours and circumstances during the first lockdown. In these change questions, participants were asked whether their behaviours and circumstances had ‘increased/were better off’, ‘stayed the same’, or ‘decreased/were worse off’ regarding studies/work, financial status, physical activity, media engagement and sleep. There were four additional questions on new activities participants had engaged in during the pandemic (making new friends, developing new hobbies, starting new voluntary work and engaging in acts of kindness) for which we asked for a yes or no response. See Tables 3, 6 and 7 for a full variable list.

### Mental health symptoms

#### Anxiety

The GAD-7^18^ includes seven Likert scale questions on the frequency of symptoms experienced, which are scored from 0 (not at all) to 3 (nearly every day). A total score of 0–4 indicates no symptoms, 5–9 mild symptoms, 10–14 moderate symptoms and 15–21 severe symptoms.

#### Depression

The PHQ-9^18^ asks nine Likert scale questions on the frequency of symptoms experienced, which are scored from 0 (not at all) to 3 (nearly every day). Total scores are categorised: 0–4 no symptoms, 5–9 mild symptoms, 10–14 moderate symptoms, 15–19 moderately severe symptoms and 20–27 severe symptoms.

#### Insomnia

The Insomnia Severity Index (ISI-3)^19^ includes three questions on sleep satisfaction, worry about sleep and how sleep interferes with daily functioning. Each question is scored between 0 (very satisfied, not at all worried/interfering) and 4 (very dissatisfied, very much worried/interfering). A total score of 7–12 indicates clinical insomnia.

#### Substance misuse

The National Institute of Drug Abuse Alcohol Smoking Substance Involvement Screening Test (NIDA-ASSIST)^20^ includes seven Likert scale questions on the frequency and impact of use for four substances: alcohol, tobacco, prescription medication and other substances. Each question is weighted differently depending on the severity of the risk examined by the item: for example, the item asking participants ‘how often have you failed to do what was normally expected of you because of your use of [first drug, second drug, etc.]’ is weighted more heavily than the question ‘In the past three months, how often have you used the substances you mentioned [first drug, second drug, etc.]?’. The average total score for all substances was used to evaluate risk: low risk (0–3.49), moderate risk (3.5–26.49) and high risk (26.5–39).

#### Suicidality

The Suicide Behaviours Questionnaire – Revised (SBQ-R)^21^ includes four questions to assess different dimensions of suicidality: lifetime suicide ideation/attempt, frequency of suicide ideation/attempt, threat of suicide attempt and future suicidal behaviour. The total score ranges from 3 to 18, and a cut-off score of ≥7 is recommended for use in non-clinical samples.

### Analysis

Statistical analyses were performed using SPSS 27.^22^

#### Handling of missing data and incomplete survey submission

A total of 1442 participants completed the T1 survey. As the survey was online, not all responses were complete (defined as surveys returned by participants who provided informed consent and engaged with the questionnaire until the very end). The survey completion rate was high (78%) among participants recruited from Prolific. However, of the participants who were recruited using convenience sampling, 716 started the survey but only 428 (60%) completed and returned the survey.

As part of the data preparation, chi-squared tests and *t*-tests were conducted to examine any differences in demographics between groups of participants who did and did not complete the baseline survey. There were no significant differences in gender composition between completers and non-completers, but there were higher rates of incomplete surveys among those returned by participants who were younger and self-identified as Asian/Asian British, Black/African/Caribbean/Black British or of other ethnicity (Supplementary Table 1 available at https://doi.org/10.1192/bjo.2022.523). At 6 months, there were also higher rates of incomplete surveys among those returned by participants who self-identified as Asian/Asian British, Black/African/Caribbean/Black British, or of mixed or other ethnicity. However, no significant differences were detected for age, gender or any of the baseline mental health outcome measures (Supplementary Table 2).

To counteract potential biases introduced through attrition (i.e. non-completion of surveys), stabilised inverse propensity scores were applied in analyses as weights.^23,24^ The propensity score referred to the conditional probability of being a completer of the survey and was calculated using three key demographic variables (age, gender and ethnicity) of all survey starters. The inverse of the propensity score was then multiplied by the marginal probability of being a survey completer. The resultant stabilised inverse probability weights (SIPW) help to balance biases introduced through attrition; this approach has the advantage of preserving the sample size of the original data and minimising the type I error rate in the estimation of variances of main effects. Two SIPWs were calculated: one for baseline and one for 6 month follow-up.

**Fig. 2:**
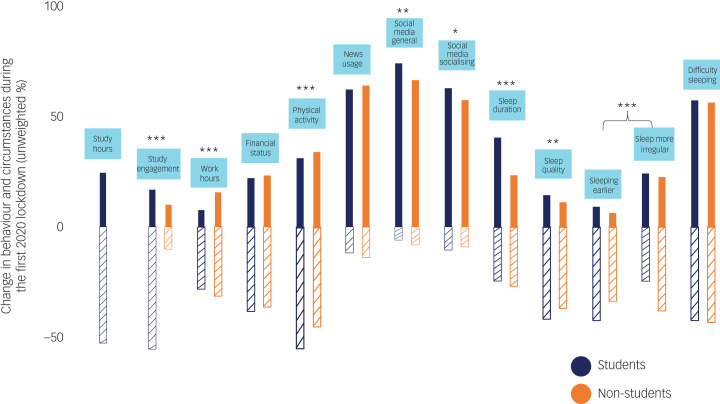
Changes in behaviours and circumstances since the lockdown at T1. The positive y-axis represents the percentage increase/improved and the negative y-axis represents percentage decrease/worsened. For sleeping earlier, the negative y-axis represents the percentage of participants who reported sleeping later. For sleep more irregular, the negative y-axis represents the percentage of participants who reported that their sleep pattern stayed the same. For difficulty sleeping, the negative y-axis represents the percentage of participants who reported not having difficulty sleeping. Only unweighted percentages are presented for easy interpretation, as weighted percentages were almost identical and the pattern of differences revealed by chi-squared (χ^2^) tests are the same. Only one χ^2^ was conducted for sleeping earlier and sleep more irregular as these were extracted from the same question, with four response options on changes in sleeping patterns (‘going to sleep at an earlier time than is usual for you’, ‘going to sleep at a later time than is usual for you’, ‘lost its regularity’ and ‘stayed about the same’). *P < 0.05, **P < 0.01, ***P < 0.001.

Unweighted and weighted analyses were performed with data from those who completed the entire survey. Weighted beta-coefficients and statistical significance were reported for all regression analyses. Unweighted descriptive statistics (percentages of self-reported changes in behaviours and circumstances) are presented in Figs 1 and 2 and the demographic variables of T2 participants shown in Table 4 for easy interpretation.

#### Cross-sectional analysis

To characterise and detect differences between groups (students and non-students), *t*-tests or chi-squared tests were conducted to compare all relevant variables. Means and standard deviations were reported for continuous variables and frequencies and percentages for categorical variables.

Regression analysis was conducted using T1 survey data. Five sets of analysis were carried out for the five mental health symptoms of interest (i.e. total scores on GAD-7, PHQ-9, ISI-3, NIDA-ASSIST and SBQ-R).

We had 45 possible predictors that could be included in the full regression model, but given the sample size of 1442 for the cross-sectional analysis and 201 for the longitudinal analysis (described in the next section), we ran univariate regression models for each individual predictor (model 1) and only included those that were significant at *P* < 0.05 in our multivariate models (model 2), to avoid overfitting.^25^

In model 2 for each set of analysis, significant univariate predictors were entered in blocks in the following order: group (students or non-students) and demographic characteristics (block 1), COVID-19 risk and exposure (block 2), and changes in behaviour and circumstance since lockdown (block 3). This allowed us to determine the specific risk(s) to young people's mental health associated with studying at university and to examine the role of changes in behaviours and circumstances due to COVID, controlling for demographics and COVID-19 risk and exposure, which were less amenable to change.

#### Longitudinal analysis

Changes in mental health symptoms over time were analysed using available data provided by student participants who completed both T1 and T2 surveys. Paired *t*-tests and chi-squared tests were used to detect these changes.

Longitudinal regression analysis was conducted using the same approach as that used for the cross-sectional analysis, but the outcomes predicted were total scores on GAD-7, PHQ-9, ISI-3, NIDA-ASSIST and SBQ-R at T2. We had a total of 50 possible predictors that could be included in the full regression model. Again, we ran univariate regression models for each individual predictor (model 1) and only included those that were significant at *P* < 0.05 in our multivariate models (model 2), to avoid overfitting.^25^ In model 2 for each set of analysis, significant univariate predictors were entered in blocks in the following order: demographic characteristics (block 1), study characteristics (block 2), COVID-19 risk and exposure (block 3), and changes in behaviour and circumstance since lockdown (block 4). In addition to models 1 and 2, a model 3 was used for each set of analysis to include the respective score at T1 as a predictor, to further adjust for baseline status.

## Results

### Participant characteristics and comparisons between students and non-students

Students sampled were predominantly UK domestic students (56%), followed by EU (26%) and other international students (16%); only 1.7% of students sampled were visiting students. At T1, 70% of students were undergraduate and 29% were postgraduate students, and most students sampled were in full-time education (86%). Few students were living on campus before lockdown (30%); after lockdown, even fewer were still on campus (14.5%). Of students sampled, 52% had exams in 2020, 25% did not have exams and 24% said they were given alternative assessments.

As presented in Table 1, the student and non-student groups shared similarities with respect to many aspects measured, but they differed in age, ethnicity and current location, with the student sample being younger, more diverse and more international compared with the non-student sample. Students were living in larger groupings and a greater proportion of them were living with friends or housemates than non-students, who more often were living with partners. Related to this, students also reported higher exposure to COVID-19 cases in their extended family and social network than non-students. Although no difference was observed in pre-existing physical condition(s) reported, a larger proportion of non-students reported moderate COVID-19 risk as defined by the UK government and a pre-existing mental health condition compared with students.

**Table 1:** Participant characteristics and group comparisons at T1

Variable	Students		Non-students		Total		Comparison *t* or χ^2^	Weighted comparison *t* or χ^2^
	Mean, *n*	s.d., %	Mean, *n*	s.d., %	Mean, *n*	s.d., %		
Age, years	23.4	6.4	27.2	4.7	25.4	20.7	***t*** = −12.07*** χ^2^ = 301.78***	−11.61*** 260.20***
20 or under	311	34.8	65	11.9	376	26.1		
21–24	360	40.3	97	17.7	457	31.7		
25–29	143	16.0	188	34.4	331	23.0		
30 or over	80	8.9	197	36.0	277	19.2		
Total	894	100	547	100	1441	100		
Gender							**χ^2^** = 0.61	0.37
Female	498	55.6	313	57.2	811	56.2		
Male	382	42.7	227	41.5	609	42.2		
Other	15	1.7	7	1.3	22	1.5		
Total	895	100	547	100	1442	100		
Ethnicity							**χ^2^** = 98.54***	89.13***
White/Caucasian	624	69.9	501	91.6	1125	78.1		
African/Caribbean/ Black British	42	4.7	10	1.8	52	3.6		
Asian/Asian British	150	16.8	17	3.1	167	11.6		
Mixed	54	6.0	17	3.1	71	4.9		
Other	23	2.6	2	0.4	25	1.7		
Total	893	100	547	100	1442	100		
Current location							**χ^2^** = 135.71***	111.93***
UK	595	66.5	510	93.2	1105	76.6		
Non-UK	300	33.5	37	6.8	337	23.4		
Total	895	100	547	100	1442	100		
Current household members^a^^,^^b^							**χ^2^** = 28.28***	25.97***
Family	662	80.2	418	83.3	1080	81.4		
Friend/housemates	91	11.0	20	4	111	8.4		
Partners	56	6.8	60	12.0	116	8.7		
Other	16	2	4	0.8	20	1.6		
Total^5^	825	100	502	100	1327	100		
Current household size^a^							**χ^2^** = 29.21***	25.23***
Alone	70	7.8	45	8.2	115	8.0		
1–2 other people	351	39.2	292	53.4	643	44.6		
3+ other people	466	52.1	209	38.2	675	46.8		
Other	8	0.9	1	0.2	9	0.6		
Total	895	100	547	100	1442	100		
Physical health condition^c^							**χ^2^** = 2.60	0.01
Yes	168	18.8	104	19.0	272	18.9		
No	709	79.2	438	80.1	1147	79.5		
Other	18	2.0	5	0.9	23	1.6		
Total	895	100	547	100	1442	100		
Mental health condition^c^							**χ^2^** = 22.16***	16.59***
Yes	210	23.5	289	34.6	399	27.7		
No	656	73.3	348	63.6	1004	69.6		
Other	29	3.2	10	1.8	39	2.7		
Total	895	100	547	100	1442	100		
Using a health tracker app^a^							**χ^2^** = 0.85	0.65
Yes	319	35.6	210	38.4	529	36.7		
No	566	63.2	336	61.4	902	62.6		
Other	10	1.1	1	0.2	11	0.8		
Total	895	100	547	100	1442	100		
Carer status^a^							**χ^2^** = 0.11	0.02
Yes	106	11.8	62	11.3	168	11.7		
No	779	87.0	482	88.1	1261	87.4		
Other	10	1.1	3	0.5	13	0.9		
Total	895	100	547	100	1442	100		
Sleep chronotype							**χ^2^** = 0.37	0.29
Morning lark	206	23.0	133	24.3	339	23.5		
Night owl	469	52.4	279	51.0	748	51.9		
Other	220	24.5	135	24.7	345	24.6		
Total	895	100	547	100	1442	100		
COVID-19 high risk^a^^,^^d^							**χ^2^** = 1.41	0.95
Yes	27	3.0	23	4.2	50	3.5		
No	862	96.5	522	95.4	1384	96.1		
Other	4	0.4	2	0.4	6	0.4		
Total	893	100	547	100	1440	100		
COVID-19 moderate risk^a^^,^^e^							**χ^2^** = 1.47****	10.53****
Yes	98	11.0	95	17.4	193	13.4		
No	785	87.9	451	82.4	1236	85.8		
Other	10	1.1	1	0.2	11	0.8		
Total	893	100	547	100	1440	100		
Tested positive for COVID-19^a^							**χ^2^** = 0.99	1.00
Yes	8	0.9	8	1.5	16	1.1		
No	884	98.8	538	98.4	1422	98.6		
Other	3	0.3	1	0.2	4	0.3		
Total	895	100	547	100	1442	100		
Tested positive for COVID-19 (family/friends)^a^							**χ^2^** = 0.79	0.55
Yes	100	11.2	70	12.8	170	11.8		
No	790	88.3	477	87.2	1267	87.9		
Other	5	0.6	0	0	5	0.3		
Total	895	100	547	100	1442	100		
Tested positive for COVID-19 (Extended family/social network)^a^							**χ^2^** = 10.56****	9.15****
Yes	302	33.7	141	25.8	443	30.7		
No	585	65.4	404	73.9	989	68.6		
Other	8	0.9	2	0.4	10	0.7		
Total	895	100	547	100	1442	100		

Unweighted frequency, percentage and χ² are reported for all variables except age, for which mean, standard deviation and *t* are reported also. Weighted comparisons are reported in the final column on the right.

***P < 0.01, ****P < 0.001.

a. χ² was calculated without the ‘other’ category, because including this category violated χ² assumptions as two cells had expected count less than 5.

b. Excluding those who answered ‘living alone’ in the current household size question.

c. Physical health problem and mental health problem refer specifically to diagnosed conditions.

d. High risk: as defined by the UK government regulation – received bone marrow or stem cell transplant in the past 6 months, or are still taking immunosuppressant medicine, received an organ transplant, severe lung condition (such as cystic fibrosis, severe asthma or severe chronic obstructive pulmonary disease (COPD)), having chemotherapy or antibody treatment for cancer, including immunotherapy, have a condition that means a very high risk of getting infections (such as severe combined immunodeficiency (SCID) or sickle cell), having an intense course of radiotherapy (radical radiotherapy) for lung cancer, taking medicine that makes them much more likely to get infections (such as high doses of steroids), having targeted cancer treatments that can affect the immune system (such as protein kinase inhibitors or PARP inhibitors), have a serious heart condition and are pregnant, had blood or bone marrow cancer (such as leukaemia, lymphoma or myeloma).

e. Moderate risk: as defined by the UK government regulation – have liver disease (such as hepatitis), age 70 or older, have a condition affecting the brain or nerves (such as Parkinson's disease, motor neurone disease, multiple sclerosis or cerebral palsy), pregnant, have a condition that means a high risk of getting infections, have a lung condition that is not severe (such as asthma, COPD, emphysema or bronchitis), taking medicine that can affect the immune system (such as low doses of steroids), have heart disease (such as heart failure), very obese (body mass index of 40 or above), have diabetes, have chronic kidney disease.

### Self-reported changes in behaviour and circumstances at T1

**Fig. 3:**
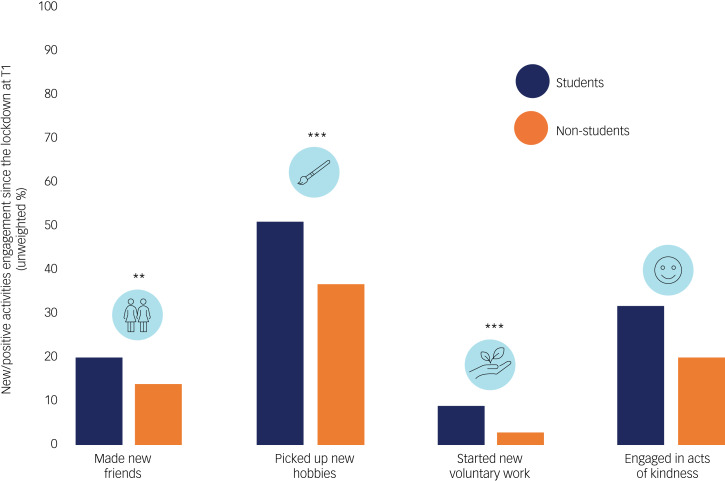
New/positive activities engagement since the lockdown at T1. Graph reporting the percentage of participants, by group, who engaged in the featured new/positive activities since the first lockdown. Only unweighted percentages are presented for easy interpretation, as weighted percentages were almost identical and the pattern of differences revealed by chi-squared tests were the same. *P < 0.05, **P < 0.01, ***P < 0.001.

Figure 2 depicts the changes in behaviours and circumstances reported by participants, and Fig. 3 shows the percentages of reported new/positive activities. In Fig. 2, the positive y-axis represents the percentage increase/improvement and the negative y-axis represents percentage decrease/worsening. Only changes are presented; the percentage that stayed the same for each category of behaviour or circumstances was not shown. Overall, there were greater percentages of both student and non-student participants reporting a reduction in study hours/engagement, work hours, financial status and physical activity since the first lockdown, compared with an increase/improvement in these aspects. Within both groups, the majority of the participants reported an increase in media engagement, including news usage and social media use for general browsing and socialising. For the sleep questions, more participants reported an increase in sleep duration but a reduction in sleep quality. There were also more participants reporting going to bed at a later time than usual and having difficulty sleeping but not necessarily a more irregular sleep pattern. Engagement in new/positive activities was reported, with between 20 and 32% reported having engaged in acts of kindness and between 37 and 51% reporting picking up new hobbies since the lockdown.

There were significant differences between student and non-student participants in these reported changes in 9 out of 12 behaviours or circumstances and engagement in three out of four new/positive activities.

### Mental health symptoms at T1

Based on responses to the GAD-7, PHQ-9, ISI-3, NIDA-ASSIST and SBQ-R (Table 2), just over 40% of participants reported moderate to severe symptoms of anxiety and depression, 25% reported clinically significant insomnia, 35% reported moderate to high risk of substance misuse and 33% reported clinically significant suicidal risk.

**Table 2:** Mental health symptoms and group comparisons at T1

Variable	Students		Non-students		Total		Comparison *t* or χ^2^	Weighted Comparison *t* or χ^2^
	Mean, *n*	s.d., %	Mean, *n*	s.d., %	Mean, *n*	s.d., %		
GAD-7								
Mean (95% CI)	8.47 (8.1−8.9)	5.93	8.66 (8.1−9.2)	6.20	8.4 (8.2−8.9)	6.03	***t*** = −0.56	−0.53
None	277	31.0	173	31.6	450	31.3	**χ^2^** = 2.40	2.20
Mild	252	28.2	147	26.9	399	27.7		
Moderate	197	22.1	109	19.9	306	21.3		
Severe	167	18.7	118	21.6	285	19.8		
Total	893	100	547	100	1440	100		
PHQ-9								
Mean (95% CI)	8.93 (8.5−9.3)	6.06	9.6 (9.0−10.2)	6.84	9.18 (8.9−9.5)	6.37	***t*** = −1.88	−1.77
None	249	27.9	167	30.6	416	28.9	**χ^2^** = 14.76***	13.04**
Mild	284	31.8	130	23.8	414	28.7		
Moderate	177	19.8	111	20.3	288	20.0		
Moderately Severe	130	14.5	85	15.6	215	14.9		
Severe	54	6.0	53	9.7	107	7.4		
Total	894	100	546	100	1440	100		
ISI-3								
Mean (95% CI)	3.63 (3.4−3.9)	3.63	3.60 (3.3−3.9)	3.72	3.62 (3.4−3.8)	3.66	***t*** = 0.13	0.09
Not clinically significant	670	74.9	411	75.1	1081	75.0	**χ^2^** = 0.01	0.02
Clinically significant	225	25.1	136	24.9	361	25.0		
Total	895	100	547	100	1442	100		
NIDA-ASSIST								
Mean (95% CI)	3.35 (3.0−3.6)	4.54	4.24 (3.8−4.6)	4.78	3.69 (3.4−3.9)	4.65	***t*** = −3.51****	−3.21***
Low Risk	609	69.0	324	59.6	933	65.4	**χ^2^** = 16.71****	13.59***
Moderate Risk	267	30.2	219	40.3	486	34.1		
High Risk	7	0.8	1	0.2	8	0.6		
Total	883	100	544	100	1427	100		
SBQ-R								
Mean (95% CI)	5.72 (5.5−5.9)	3.12	6.08 (5.8−6.4)	3.68	5.86 (5.7−6.0)	3.34	***t*** = −1.89	−1.66
Not clinically significant	608	68.7	350	65.1	958	67.3	**χ^2^** = 2.02	1.53
Clinically significant	277	31.3	188	34.9	465	32.7		
Total	885	100	538	100	1423	100		

Unweighted mean values, 95% confidence intervals, standard deviations, *t* are reported for each outcome measure, alongside number and percentage of cases in each score category for easy interpretation. Weighted comparisons are reported in the final column on the right.

GAD-7 (anxiety) scores: 0–4, none; 5–9, mild; 10–14, moderate; 15+, severe. PHQ-9 (depression) scores: 0–4, none; 5–9, mild; 10–14, moderate; 15–19, moderately severe; 20+, severe. ISI-3 (insomnia) scores: 0–6, not clinically significant; 7+, clinically significant. NIDA-ASSIST (substance misuse) scores: 0–3 (0–3.49), low risk; 4–26 (3.5–26.49), moderate risk; 27+ (26.5 or above), high risk. SBQ-R (suicidality) scores: 0–6, not clinically significant; 7+ (7 or above) clinically significant.

**P < 0.05, ***P < 0.01, ****P < 0.001.

There were no significant differences between student and non-student participants in their levels of anxiety, depression, insomnia and suicidality at T1, with the exception of substance misuse risk. The mean NIDA-ASSIST score was higher among non-student than student participants. The proportions of participants reporting moderate risk of substance misuse or severe depression were higher for non-student compared with student participants.

### Risk markers of mental health symptoms at T1

Table 3 shows regression coefficients of all risk markers included in model 2 for all five mental health outcome measures.

**Table 3:** Risk markers for anxiety, depression, insomnia, substance misuse and suicidality at T1, based on model 2

Variable	Anxiety (GAD-7)		Depression (PHQ-9)		Insomnia (ISI-3)		Substance misuse (NIDA-ASSIST)		Suicidality (SBQ-R)	
	*β*	B	*β*	B	*β*	B	*β*	B	*β*	B
Demographic characteristics										
Student status							**−0**.108***	**−1**.**037**		
Age	**−0**.098****	**−0**.**100**	**−0**.100****	**−0**.**107**	0.034	0.021	0.023	0.018	**−0**.138****	**−0**.**078**
Gender: female	0.046	0.560	0.003	0.044	0.000	0.002	**−0**.106****	**−0**.**997**		
Gender: male									0.045	0.305
Gender: other									**0**.057**	**1**.**535**
White/Caucasian							−0.005	−0.053		
Black/Black British										
Asian/Asian British					0.029	0.318	**−0**.084**	**−1**.**185**		
Mixed ethnicity					0.035*	0.548				
Currently UK-based							**−0**.146****	**−1**.**596**		
Changed location (past 3 months)									0.048	0.389
Living with: family							−0.045	−0.539		
Living with: friends/housemates							**0**.076**	**1**.**270**		
Living with: partner										
Living with: other										
Living alone										
Living with 1–2 others										
Living with 3+ other										
Physical health condition	−0.010	−0.158	−0.008	−0.125	−0.035	−0.330			0.019	0.165
Mental health condition	**0**.238****	**3**.**185**	**0**.254****	**3**.**587**	**0**.071****	**0**.**578**	**0**.170****	**1**.**755**	**0**.436****	**3**.**238**
Using health app	0.025	0.314			**0**.054***	**0**.**408**				
Carer	**0**.082***	**1.546**	**0**.096****	**1**.**909**	**0**.037**	**0**.**424**				
Chronotype: morning lark			−0.015	−0.231	−0.035	−0.301				
Chronotype: night owl			0.026	0.327	−0.001	−0.010			0.020	0.134
**Δ*R^2^***	0.120		0.130		0.093		0.087		0.229	
**Δ*F***	26.699****		25.108****		11.373****		11.554****		49.245****	
COVID-19 risk and exposure										
COVID high risk	0.022	0.725	0.028	0.981	0.017	0.334	0.035	0.899	0.006	0.115
COVID moderate risk	0.021	0.372	0.022	0.410	0.026	0.283			0.010	0.094
Tested positive							**0**.072**	**3**.**214**		
Tested positive – close family	0.018	0.340	0.022	0.428	0.005	0.059				
Tested positive – extended network	0.045	0.591	0.024	0.336	0.032	0.251				
**Δ*R^2^***	0.010		0.010		0.106		0.007		0.001	
**Δ*F***	3.480***		3.447***		4.107***		4.122**		0.654	
Changes since lockdown										
Work hours	−0.036	−0.363	−0.032	−0.335	−0.009	−0.056	−0.051	−0.392	−0.047	−0.263
Physical activity	−0.018	−0.123	−**0**.078***	−**0**.**554**	−0.012	−0.049				
News consumption										
Social media use (general browsing)	0.028	0.285	0.025	0.265	0.002	0.012				
Social media use (socialising)										
Financial status	−**0**.136****	−**1**.**076**	−**0**.133****	−**1**.**118**	−0.020	−0.094	−**0**.073**	−**0**.**447**	−**0**.061**	−**0**.**267**
Sleep duration			−0.004	−0.027						
Sleep quality	−0.019	−0.167	−0.033	−0.301	−0.022	−0.117				
Sleeping earlier			0.014	0.328	0.010	0.132			0.013	0.103
Sleeping later					0.004	0.029				
Sleep pattern more irregular	**0**.109****	**1**.**537**	**0**.117****	**1**.**751**	**0**.047**	**0**.**406**	**0**.077***	**0**.**846**		
Difficulty sleeping	**0**.299****	**3**.**642**	**0**.319****	**4**.**105**	**0**.767****	**5**.**668**	0.039	0.363	**0**.147****	**0**.**994**
New friends										
New hobbies	0.028	0.339								
New voluntary work										
Acts of kindness					0.006	0.046				
**Δ*R^2^***	0.150		0.173		0.567		0.020		0.030	
**Δ*F***	30.243****		32.552****		190.296****		6.095****		11.853****	
**Full model – R^2^**	0.280		0.314		0.673		0.114		0.260	
**Full model – F**	25.135****		26.519****		94.144****		9.288****		31.245****	

Model 2 is a multivariate hierarchical linear regression model, wherein all significant univariate predictors from model 1 (*P* < 0.05) were included and entered by block (‘Demographic characteristics’ → ‘COVID-19 risk and exposure’ → ‘Changes since lockdown’). Results from the weighted analysis are presented to show patterns of significance (highlighted in bold). *ΔR^2^* (change) and *ΔF* (change) are presented for each block of predictors. *R^2^* and *F* are presented for the full model. A full list of predicting variables is presented in the first column on the left. Empty boxes exist because only significant predicting variables in model 1 were included in model 2. All categorical variables were dummy coded, e.g. gender: female (1, = female, 0 = not female), except several of the ‘changes since lockdown’ variables (from work hours to financial status), which were coded as 1 = increase/better off, 0 = stayed about the same, −1 = decrease/worse off. Multicollinearity, as indicated by variance inflation factor (VIF) > 4, was detected between predictors within models. Individual predictors were removed to maintain model stability and accuracy (anxiety model, gender: male; depression model, gender: male; insomnia model, gender: male and ethnicity: White/Caucasian; substance misuse model, gender: male.

*β*, standardised beta coefficient; Β, unstandardised beta coefficient.

* *P* = 0.05, **P < 0.05, ***P < 0.01, ****P < 0.001.

### Anxiety

The significant risk markers of anxiety were younger age, having a previously diagnosed mental health condition, being a carer, worse financial status, more irregular sleep pattern and increased difficulty sleeping. The co-efficient of determination (R^2^) for model 2 was 0.280.

### Depression

The significant risk markers of depression were younger age, having a previously diagnosed mental health condition, being a carer, reduced physical activity, worse financial status, more irregular sleep pattern and increased difficulty sleeping. The R^2^ for model 2 was 0.314.

### Insomnia

The significant risk markers of insomnia were having a previously diagnosed mental health condition, using a health tracker app, being a carer, more irregular sleep pattern and increased difficulty sleeping. The R^2^ for model 2 was 0.673.

### Substance misuse

The significant risk markers of substance misuse were not being a student, gender (not female), ethnically not Asian/Asian British, not currently in the UK, living with friends or housemates, having a previously diagnosed mental health condition, having tested positive for COVID-19, worse financial status and more irregular sleep pattern. The R^2^ for model 2 was 0.114.

### Suicidality

The significant risk markers of suicidality were younger age, gender (other), having a previously diagnosed mental health condition, worse financial status and increased difficulty sleeping. The R^2^ for model 2 was 0.260.

### Longitudinal analysis

#### Participant characteristics and attrition at T2

Of the 365 student participants recruited from a single university who consented to being recontacted, 201 participants completed the second survey at T2 (44.9% attrition). Table 4 presents these participants’ characteristics based on available data.

**Table 4:** Participant characteristics at T2

Variable	Students	
	Mean, *n*	s.d., %
Age (years)	23.85	8.15
20 or under	81	42.6
21–24	60	31.6
25–29	30	15.8
30 or over	19	10
Total	190	100
Gender		
Female	129	30.9
Male	59	67.5
Other	3	1.6
Total	191	100
Ethnicity		
White/Caucasian	132	69.1
African/Caribbean/ Black British	6	3.1
Asian/Asian British	38	19.9
Mixed	11	5.8
Other	3	1.6
Total	190	100
Current location		
UK	162	85.3
Non-UK	28	14.7
Total	190	100
Current household members		
Family	139	79.0
Friend/housemates	18	10.2
Partners	12	6.8
Other	7	4.0
Total^a^	176	100
Current household size		
Alone	14	7.4
1–2 other people	77	40.5
3+ other people	99	52.1
Other	0	0
Total	190	100
Physical health condition^b^		
Yes	46	24.1
No	142	74.3
Other	2	1.0
Total	190	100
Mental health condition^b^		
Yes	53	27.7
No	131	68.6
Other	6	3.1
Total	190	100
Using a health tracker app		
Yes	72	37.7
No	118	61.8
Other	0	0
Total	190	100
Carer status		
Yes	15	7.9
No	173	91.1
Other	2	1.1
Total	190	100
Sleep chronotype		
Morning lark	53	27.9
Night owl	83	43.7
Other	54	28.4
Total	190	100
COVID-19 high risk^c^		
Yes	3	1.6
No	187	98.4
Other	0	0
Total	190	100
COVID-19 moderate risk^d^		
Yes	26	13.7
No	163	85.8
Other	1	0.5
Total	190	100
Tested positive for COVID-19		
Yes	1	0.5
No	188	98.9
Other	1	0.5
Total	190	100
Tested positive for COVID-19 (family/friends)		
Yes	15	7.9
No	175	92.1
Other	0	0
Total	190	100
Tested positive for COVID-19 (extended family/social network)		
Yes	86	45.0
No	104	54.5
Other	0	0
Total	190	100
Additional student characteristics		
Student type		
Home/UK	140	73.7
EU	25	13.2
International	25	13.2
Visiting student	0	0
Other	0	0
Total	190	100
Study level		
Undergraduate^e^	123	65.8
Postgraduate	64	4.2
Other	0	0
Total	187	100
Course type		
Full-time	169	88.9
Part-time	21	11.1
Other	0	0
Total	190	100
Exams this academic year		
Yes	76	40.9
No	49	26.3
Other	61	32.8
Total	186	100

Unweighted frequency and percentage are reported for all variables except age, for which mean, standard deviation and *t* are reported also.

a. Excluding those who answered ‘living alone’ in the current household size question.

b. Physical health problem and mental health problem refer specifically to diagnosed conditions.

c. High risk: as defined by UK government regulations – received bone marrow or stem cell transplant in the past 6 months, or are still taking immunosuppressant medicine, received an organ transplant, severe lung condition (such as cystic fibrosis, severe asthma or severe chronic obstructive pulmonary disease (COPD)), having chemotherapy or antibody treatment for cancer, including immunotherapy, have a condition that means a very high risk of getting infections (such as SCID or sickle cell), having an intense course of radiotherapy (radical radiotherapy) for lung cancer, taking medicine that makes them much more likely to get infections (such as high doses of steroids), having targeted cancer treatments that can affect the immune system (such as protein kinase inhibitors or PARP inhibitors), have a serious heart condition and are pregnant, had blood or bone marrow cancer (such as leukaemia, lymphoma or myeloma).

d. Moderate risk: as defined by UK government regulations – have liver disease (such as hepatitis), aged 70 or older, have a condition affecting the brain or nerves (such as Parkinson's disease, motor neurone disease, multiple sclerosis or cerebral palsy), pregnant, have a condition that means a high risk of getting infections, have a lung condition that is not severe (such as asthma, COPD, emphysema or bronchitis), taking medicine that can affect the immune system (such as low doses of steroids), have heart disease (such as heart failure), very obese (body mass index of 40 or above), have diabetes, have chronic kidney disease.

e. Undergraduate included bachelor's and foundation students.

#### Changes in mental health symptoms between T1 and T2

There was a significant reduction in reported anxiety and depression between T1 and T2, as shown in Table 5. The percentage of participants reporting any symptoms of anxiety reduced from 72.1% at T1 to 59.3% at T2. There was little change in the percentage of participants experiencing mild and moderate anxiety symptoms, but between T1 and T2 the percentage of participants reporting severe symptoms fell from 18.4% to 6.9%. Similarly, the percentage of participants reporting any depression symptoms fell from 69.8% to 61.4%. For depression, there was little change in the percentage of participants experiencing severe symptoms, whereas the number of participants experiencing mild symptoms fell by 4.8% and the percentage of participants experiencing moderately severe symptoms fell by 3.2%.

**Table 5:** Mental health symptoms of follow-up responders and comparisons between T1 and T2

Variable^a^	T1		T2		Comparison	Weighted comparison
	Mean, *n*	s.d., %	Mean, *n*	s.d., %	*t* mean difference (95% CI)	*t* mean difference (95% CI)
GAD-7						
Mean	8.65	5.93	6.68	5.04	5.14**** **1.97 (1.22**−**2.73)**	4.76**** **1.91 (1.12**−**2.71)**
None	53	27.9	77	40.7		
Mild	55	28.9	56	29.6		
Moderate	47	24.7	43	22.8		
Severe	35	18.4	13	6.9		
Total	190	100	189	100		
PHQ-9						
Mean	8.18	5.70	7.45	5.92	2.08**, **0.73 (0.04**−**1.42)**	1.98*, **0.73 (0.00**−**1.47)**
None	57	30.2	73	38.6		
Mild	68	36.0	59	31.2		
Moderate	28	14.8	27	14.3		
Moderately Severe	26	13.8	20	10.6		
Severe	10	5.3	10	5.3		
Total	189	100	189	100		
ISI-3						
Mean	3.64	3.45	3.11	3.31	1.86, 0.53 (-0.03−1.10)	1.50, 0.45 (−0.15−1.05)
Not clinically significant	146	76.8	158	83.2		
Clinically significant	44	23.2	32	16.8		
Total	190	100	190	100		
NIDA-ASSIST						
Mean	2.18	2.71	2.29	3.03	−0.70, −0.11 (−0.41−0.20)	−0.67, −0.11(−0.43−0.21)
Low Risk	151	80.3	149	78.8		
Moderate Risk	37	19.7	40	21.2		
High Risk	0	0	0	0		
Total	188	100	189	100		
SBQ-R						
Mean	5.92	2.91	6.03	3.34	−0.77, −0.11 (−0.40−0.17)	−0.71, −0.11 (−0.41−0.19)
Not clinically significant	122	64.6	119	63.0		
Clinically significant	67	35.4	70	37.0		
Total	189	100	189	100		

Unweighted mean values and standard deviations are reported for each outcome measure, with *t*-values, mean differences between T1 and T2, and 95% confidence intervals of the differences. The number and percentage of cases in each score category are also reported for easy interpretation. Weighted comparisons are reported in the final column on the right.

a. GAD-7 (anxiety) scores: 0–4, none; 5–9, mild; 10–14, moderate; 15+, severe. PHQ-9 (depression) scores: 0–4, none; 5–9, mild; 10–14, moderate; 15–19, moderately severe; 20+, severe. ISI-3 (insomnia) scores: 0–6, not clinically significant; 7+, clinically significant. NIDA-ASSIST (substance misuse) scores: 0–3 (0–3.49), low risk; 4–26 (3.5–26.49), moderate risk; 27+ (26.5 or above), high risk. SBQ-R (suicidality) scores: 0–6, not clinically significant; 7+, clinically significant.

*P = 0.05, **P < 0.05, ****P < 0.001.

There were no significant differences in reported insomnia, substance misuse or suicidality between T1 and T2, even though the percentage of participants reporting clinically significant insomnia dropped from 23.2% to 16.8% and the percentage with clinically significant suicide risk rose from 35.4% to 37%. There were no participants reporting high-risk substance misuse at either T1 or T2, but the percentage reporting moderate risk increased from 19.7% to 21.2%.

#### Risk factors for mental health symptoms at T2

##### Anxiety

The only significant risk factor for anxiety was having difficulty sleeping at T1. The R^2^ for model 2 was 0.121 (Table 6). After adjusting for baseline status (see model 3 results in Table 7), T1 anxiety score was the only significant risk factor. The R^2^ for model 3 was 0.331.

**Table 6:** Risk factors for anxiety, depression, insomnia, substance misuse and suicidality at T2, based on model 2

Variable	Anxiety (GAD-7)		Depression (PHQ-9)		Insomnia (ISI-3)		Substance misuse (NIDA-ASSIST)		Suicidality (SBQ-R)	
	*β*	B	*β*	B	*β*	B	*β*	B	*β*	B
Demographic characteristics										
Age										
Gender: female										
Gender: male										
Gender: other										
White/Caucasian										
Black/Black British										
Asian/Asian British										
Mixed ethnicity										
Currently UK-based										
Changed location (past 3 months)										
Living with: family										
Living with: friends/housemates										
Living with: partner										
Living with: other										
Living alone										
Living with 1–2 others										
Living with 3+ other										
Physical health condition										
Mental health condition										
Using health app										
Carer										
Chronotype: morning lark							**−0**.180**	**−1**.**230**		
Chronotype: night owl										
**Δ*R^2^***							**0**.**040**			
**Δ*F***							**6**.721**			
Student characteristics										
Home student or not										
UG or PG										
FT or PT										
Exams this year										
**Δ*R^2^***										
**Δ*F***										
COVID-19 risk and exposure										
COVID high risk			0.131	6.597						
COVID moderate risk										
Tested positive										
Tested positive – close family										
Tested positive – extended network					0.129	0.850				
**Δ*R^2^***			**0**.**026**		**0**.**024**					
**Δ*F***			**4**.209**		**4**.013**					
Changes since lockdown										
Study hours	−0.128	−0.793	−0.128	−0.932	−0.122	−0.499				
Study engagement	−0.040	−0.274	−0.087	−0.698						
Work hours										
Physical activity									**−0**.176**	**−0**.**661**
News consumption										
Social media use (general browsing)	0.077	0.680							0.129	0.759
Social media use (socialising)										
Financial status	−0.109	−0.668	**−0**.152**	**−1**.**125**	−0.123	−0.514			**−0**.156**	**−0**.**663**
Sleep duration										
Sleep quality										
Sleeping earlier										
Sleeping later										
Sleep pattern more irregular	0.095	1.175	0.045	0.655	0.060	0.483				
Difficulty sleeping	**0**.173**	**1**.**779**	**0**.205***	**2**.**462**	**0**.266***	**1**.**796**	**0**.201***	**1**.**260**	0.104	0.715
New friends										
New hobbies										
New voluntary work										
Acts of kindness										
**Δ*R^2^***	**0**.**121**		**0**.**129**		**0**.**129**		**0**.**040**		**0**.**103**	
**Δ*F***	**3**.556***		**4**.766****		**6**.011****		**6**.915***		**4**.536***	
**Full model – R^2^**	**0**.**121**		**0**.**155**		**0**.**154**		**0**.**080**		**0**.**103**	
**Full model – F**	**3**.556***		**4**.756****		**5**.711****		**6**.942***		**4**.536***	

Model 2 is a multivariate hierarchical linear regression model, wherein all significant univariate predictors from model 1 (*P* < 0.05) were included and entered by block (‘Demographic characteristics’ → ‘Student characteristics’ → ‘COVID-19 risk and exposure' → ‘Changes since lockdown’). Results from the weighted analysis are presented to show patterns of significance (highlighted in bold). *ΔR^2^* (change) and *ΔF* (change) are presented for each block of predictors. *R^2^* and *F* are presented for the full model. A full list of predicting variables is presented in the first column on the left. Empty boxes exist because only significant predicting variables in model 1 were included in model 2. *β*, standardised beta coefficient; Β, unstandardised beta coefficient; FT, full-time; PG, postgraduate; PT, part-time; UG, undergraduate.

**P < 0.05, ***P < 0.01, ****P < 0.001.

**Table 7:** Risk factors for anxiety, depression, insomnia, substance misuse and suicidality at T2, based on model 3

Variable	Anxiety (GAD-7)		Depression (PHQ-9)		Insomnia (ISI-3)		Substance misuse (NIDA-ASSIST)		Suicidality (SBQ-R)	
	*β*	B	*β*	B	*β*	B	*β*	B	*β*	B
**T1 value**	**0**.533****	**0**.**452**	**0**.642****	**0**.**655**	0.215**	**0**.**207**	**0**.733****	**0**.**818**	**0**.786****	**0**.**918**
**ΔR^2^**	**0**.**321**		**0**.**440**		**0**.**112**		**0**.**558**		**0**.**649**	
**ΔF**	**75**.765****		**126**.275****		**20**.330****		**200**.271****		**295**.564****	
Demographic characteristics										
Age										
Gender: female										
Gender: male										
Gender: other										
White/Caucasian										
Black/Black British										
Asian/Asian British										
Mixed ethnicity										
Currently UK-based										
Changed location (past 3 months)										
Living with: family										
Living with: friends/housemates										
Living with: partner										
Living with: other										
Living alone										
Living with 1–2 others										
Living with 3+ others										
Physical health condition										
Mental health condition										
Using health app										
Carer										
Chronotype: morning lark							−0.070	−0.479		
Chronotype: night owl										
**Δ*R^2^***							0.005			
**Δ*F***							1.722			
Student characteristics										
Home student or not										
UG or PG										
FT or PT										
Exams this year										
**Δ*R^2^***										
**Δ*F***										
COVID-19 risk and exposure										
COVID high risk			0.084	4.254						
COVID moderate risk										
Tested positive										
Tested positive – close family										
Tested positive – extended network					0.125	0.826				
**Δ*R^2^***			0.007		0.017					
**Δ*F***			1.969		3.207					
Changes since lockdown										
Study hours	−0.046	−0.285	−0.009	−0.066	−0.130	−0.532				
Study engagement	−0.025	−0.170	−0.028	−0.228						
Work hours										
Physical activity									−**0**.106**	−**0**.**398**
News consumption										
Social media use (general browsing)	0.045	0.396							0.031	0.183
Social media use (socialising)										
Financial status	−0.052	−0.326	−0.077	−0.572	−0.115	−0.479			−0.054	−0.228
Sleep duration										
Sleep quality										
Sleeping earlier										
Sleeping later										
Sleep pattern more irregular	0.010	0.129	−0.020	−0.288	0.036	0.290				
Difficulty sleeping	−0.008	−0.082	−0.017	−0.207	0.132	0.891	0.005	0.032	−0.031	−0.213
New friends										
New hobbies										
New voluntary work										
Acts of kindness										
**Δ*R^2^***	0.010		0.008		0.050		0.000		0.015	
**Δ*F***	0.390		0.446		2.394*		0.009		1.786	
**Full model – R^2^**	**0**.**331**		**0**.**455**		**0**.**180**		**0**.**563**		**0**.**665**	
**Full model – F**	**10**.911****		**18**.429****		**5**.703****		**67**.212****		**61**.706****	

Model 3 is a multivariate hierarchical linear regression model, wherein all significant univariate predictors from model 1 (*P* < 0.05) were included and entered by block (‘Demographic characteristics’ → ‘Student characteristics’ → ‘COVID-19 risk and exposure’ → ‘Changes since lockdown’), after values of the outcome measures at T1 were entered. Results from the weighted analysis are presented to show patterns of significance (highlighted in bold). *ΔR^2^* (change) and *ΔF* (change) are presented for each block of predictors. *R^2^* and *F* are presented for the full model. A full list of predicting variables is presented in the first column on the left. Empty boxes exist because only significant predicting variables in model 1 were included in model 2.

*β*, standardised beta coefficient; Β, unstandardised beta coefficient; FT, full-time; PG, postgraduate; PT, part-time; UG, undergraduate.

*P = 0.05, **P < 0.05, ****P < 0.001.

##### Depression

The only significant risk factors for depression were worse financial status and difficulty sleeping at T1. The R^2^ for model 2 was 0.155. In model 3, T1 depression score was the only significant risk factor. The R^2^ for model 3 was 0.455.

##### Insomnia

The only significant risk factor for insomnia was difficulty sleeping at T1. The R^2^ for model 2 was 0.154. In model 3, T1 insomnia score was the only significant risk factor. The R^2^ for model 3 was 0.180.

##### Substance misuse

The significant risk factors for substance misuse were not being a ‘morning lark’ and difficulty sleeping at T1. The R^2^ for model 2 was 0.080. In model 3, T1 substance misuse score was the only significant risk factor. The R^2^ for model 3 was 0.563.

##### Suicidality

The significant risk factors for suicidality were reduced physical activity and worse financial status at T1. The R^2^ for model 2 was 0.103. In model 3, T1 suicidality scores and reduced physical activity were significant risk factors. The R^2^ for model 3 was 0.665.

## Discussion

### Main findings

University students and young adults reported high levels of mental health problems even after the first lockdown in the UK as they resumed some form of ‘normality’. More than a third reported moderate to severe symptoms of anxiety and depression and risk of substance misuse. Over a quarter reported experiencing insomnia and clinically significant suicidal thoughts and behaviour. These rates approximated those reported by a previous US study^2^ but were higher than those reported in other large-scale studies conducted in China, France and the UK during the early stage of the pandemic.^1,3,5^ In contrast to the Italian finding that levels of depression rapidly returned to pre-pandemic levels once restrictions were lifted,^11^ the T1 data of this study suggested that the lockdown had a sustained impact on mental health beyond the duration of the restrictions. That said, we acknowledge that mental health problems in student cohorts have been an issue prior to the pandemic. The lockdown should not be considered to be the sole contributor to the high prevalence.

When a subset of university students was reassessed 6 months later – just as the UK entered the third lockdown – we saw significant reductions in symptoms of anxiety and depression. These changes suggest possible emotional adaptations as the situation evolved.^26^ However, some mental health issues appeared to be more ingrained than others; we did not see significant reductions in insomnia, substance misuse or suicidal risk, with 16.8% still presenting with clinically significant insomnia, 21.2% with moderate risk of substance misuse and 37% with clinically significant suicide risk at T2. The differential trajectories of different mental health symptoms suggest that university well-being services may benefit from a stronger focus on addressing irregularities in sleep–wake patterns, risky behaviours and suicide prevention as young people navigate through cycles of lockdown and lifting of restrictions.

Overall, despite differences in demographics and changes in behaviours and circumstances, university students and their broadly age-matched counterparts in this study were remarkably similar in their reported levels of mental health symptoms. Much of the risk associated with mood and sleep regulation and with suicidality during the pandemic was shared among young people regardless of their student status, suggesting an opportunity to cross-fertilise lessons learned across university and non-university settings for the development of better prevention and treatment strategies.

However, risk factors identified for each of the five mental health symptoms examined varied depending on the analytical approach used. In our cross-sectional models at T1, COVID risk and exposure were not the most significant risk markers of mental health symptoms. Instead, demographic characteristics and behavioural and circumstantial changes during lockdown were more significant and consistent risk markers across outcome measures. The most consistent of all were younger age, pre-existing mental health condition(s), being a carer, worse financial status, having a more irregular sleep pattern and increased difficulty sleeping. The total amounts of variance explained by our cross-sectional models using T1 data were 28% for anxiety, 31% for depression, 67% for insomnia, 11% for substance misuse and 26% for suicidality. In our longitudinal models using data from a more homogenous subgroup of university students with a 6-month time gap between predictors and outcomes, the total amounts of variance explained were reduced to 12%, 16%, 15%, 8% and 10% for the respective outcome measures of interest. In these longitudinal models, the range of significant predictors reduced, and the most consistent predictors of mental health symptoms at T2 were worse financial status and having difficulty sleeping since lockdown. These findings add to the existing literature on the mental health risk associated with financial pressure.^27,28^ They are also consistent with the view that circadian misalignment and sleep difficulties were common consequences of the pandemic that negatively affected core aspects of well-being and contributed to the aggravation of pre-existing psychological problems.^29^

The amount of variance explained rebounded and increased to 33% for anxiety, 46% for depression, 18% for insomnia, 56% for substance misuse and 67% for suicidality when the regression models included baseline values of the outcome measures of interest among the predictors. Such jumps in explanatory power suggest a history effect, with previous symptoms being the strongest predictor of future symptoms. The change in *R^2^* for models with baseline measures only ranged from 11–65%. This history effect reflects the inherent nature of mental health states, which tend to unfold over time, evolving and changing over the lifespan.^30^ It also underlines the importance of identifying modifiable risk factors for effective prevention and early intervention.^31^ In this study, we found that worse financial status and difficulty sleeping prospectively predicted more than one mental health symptom – and, having adjusted for baseline mental health states, reduced physical activity remained a significant risk predictor of greater suicidality. These findings are consistent with those of other studies with more representative adult samples^32^ and suggest that, in addition to providing enhanced support to vulnerable subgroups, greater efforts in maintaining promoting physical activity and ensuring financial stability throughout a generally difficult time could help to mitigate mental health risks.

### Strengths and limitations

Currently, there is little published information on mental health symptoms after the initial wave of lockdown in January to March 2020. This cross-sectional and longitudinal study extends our understanding by examining the continuous impact of the pandemic on the mental health of university students and identifying the risk markers and predictors of mental health symptoms. We included a non-student comparison group to assess the generalisability of risks from young people at university to those not in higher education. We used validated measures to assess a range of reported mental health symptoms: anxiety, depression, insomnia, substance misuse and suicidality. This allowed us to establish a mental health profile, identify common risk factors and note any differential effects of the pandemic on individual symptoms over time.

Like other studies carried out under COVID restrictions, this study was an online survey and had several methodological limitations. First, our participants were recruited through both convenience sampling methods and a research participant platform to achieve the needed sample size for our planned analyses. We restricted our participant recruitment to students attending UK universities and young adults living in the UK; however, responses received included some from visiting students who were in the UK on study abroad and from overseas students studying in the UK who had returned to their home countries at the time of the study. These responses, although they reflect the international nature of the university setting, may have introduced variations in responses owing to differences in conditions and public health measures adopted in different countries. Further, self-selection biases were possible in that young people with greater interest in mental health were more likely to take part in the survey, although the demographic profile of our participants approximated that of the whole UK university student cohort (Supplementary Table 1) and the prevalence rates of mental health symptoms reported in the current study were comparable with those of published studies with larger and more representative samples.^1–3^

Second, for the longitudinal analysis, we followed up only a subset of university students who gave us permission to do so. Attrition at 6 months was 44%, with 201 participants providing T1 and T2 data for the longitudinal analysis, despite incentives. The T2 sample was also ethnically less diverse than T1. It is unclear whether ‘survey fatigue’ and the stress associated with the emerging situation were determining factors.^33^ Poor response rates and high attrition seem characteristic of online surveys conducted during the pandemic, particularly among students: the Office for National Statistics conducted a survey in collaboration with the National Students Union in November 2020 and received a 2% response rate.^34^ Given the small sample size and the high attrition rate, the findings of the current study should be interpreted with caution even though the weighted and unweighted analyses resulted in similar patterns of findings.

Third, all measures of outcomes and risk factors were self-reported. Although validated measures were used, the responses received were subject to possible memory and reporting biases. With established cut-off scores, the scores on our outcome measures could be interpreted for potential clinical significance; however, they are no replacement for clinical diagnosis.

Finally, the current study only included two waves of data collection. To truly capture the continued impact of the pandemic on young people's mental health, more resources are required to invest in longitudinal studies with more assessments and longer follow-up periods.

## Implications

A considerable proportion of university students and young adults not in higher education experienced mental health symptoms following the first UK lockdown. Although there was a reduction in reported symptoms between summer 2020 and winter 2021, rates of mental health symptoms remained elevated. The current study highlights markers for identifying those with more severe mental health symptoms and risk factors for predicting poorer mental health.

The significance of behavioural and circumstantial risk factors should prompt universities to consider a more proactive approach in mitigating financial pressures and in promoting positive health behaviours (e.g. physical activity, sleep) that can help counteract poor mental health. Similarities between risk factors for students and young people not in higher education indicate that intervention and prevention measures available for each group may be evaluated and applied to both populations for greater generalisability.

## Data Availability

The data that support the findings of this study are available on request from the corresponding author, N.K.Y.T. The data are not publicly available owing to ethical restrictions e.g. their containing information that could compromise the privacy of research participants.

## Supplementary material

For supplementary material accompanying this paper visit http://doi.org/10.1192/bjo.2022.523. click here to view supplementary material
