# Supplementary material for: Pandemic and student mental health: mental health symptoms among university students and young adults after the first cycle of lockdown in the UK
Source: BJPsych Open. 2022 Jul 26;8(4):e138. doi: 10.1192/bjo.2022.523 (PMC9345288; doi:10.1192/bjo.2022.523)
Supplement: Supplementary file 1 [file bjosup.zip › S2056472422005233sup001.docx]

# Supplementary Material

| **Supplementary Table 1:** Baseline demographic comparisons between complete and incomplete cases at T1 | | | | | | |
| --- | --- | --- | --- | --- | --- | --- |
| **Key demographics at T1** | **2019-2020 HESA data**  **for reference^1^** | **Complete cases**  **mean (SD) or % n** | | **Incomplete cases**  **mean (SD) or % n** | | $\boldsymbol{\chi}$**2/*t*-value** |
| **Age** | n/a | 24.85 (6.074) | 1441 | 23.71 (6.587) | 292 | 2.89 ** |
| **20 or under** | 40% | 26.1% | 376 | 37.3% | 109 |  |
| **21-24 years** | 29% | 31.7% | 457 | 34.9% | 102 |  |
| **25-29 years** | 11% | 23.0% | 331 | 15.1% | 44 |  |
| **30 years or over** | 20% | 19.2% | 277 | 12.7% | 37 |  |
| **Gender ^2^** |  |  |  |  |  | 1.46 |
| Female | 57% | 56.2% | 811 | 60.1% | 175 |  |
| Male | 43% | 42.2% | 609 | 38.5% | 112 |  |
| Other | 0% | 1.5% | 22 | 1.4% | 4 |  |
| **Ethnicity** |  |  |  |  |  | 43.09*** |
| White/Caucasian | 74% | 78.1% | 1125 | 63.0% | 182 |  |
| Black/African/Caribbean/Black British | 8% | 3.6% | 52 | 4.2% | 12 |  |
| Asian/Asian British | 12% | 11.6% | 167 | 26.0% | 75 |  |
| Mixed | 4% | 4.9% | 71 | 4.8% | 14 |  |
| Other | 2% | 1.7% | 25 | 2.1% | 6 |  |

Notes:

1. HESA = Higher Education Statistics Agency, UK. Data source: <https://www.hesa.ac.uk/data-and-analysis/students/whos-in-he> (retrieved 24th Jan 2022)
2. $\chi$2 was calculated for only Male and Female categories, excluding ‘Other’ as including all gender categories violated $\chi$2 assumptions as 1 cells had a count less than 5.
3. * *p* < 0.05, ** *p* <0.01, *** *p* < 0.001
4. n/a = not available
