# Supplementary material for: Pandemic and student mental health: mental health symptoms among university students and young adults after the first cycle of lockdown in the UK
Source: BJPsych Open. 2022 Jul 26;8(4):e138. doi: 10.1192/bjo.2022.523 (PMC9345288; doi:10.1192/bjo.2022.523)
Supplement: Supplementary file 1 [file bjosup.zip › S2056472422005233sup002.docx]

**Supplementary Material**

| **Supplementary Table 2:** Baseline demographic and outcome comparisons between university students who completed T1 survey only and those who completed both T1 and T2 surveys | | | | | | | |
| --- | --- | --- | --- | --- | --- | --- | --- |
| **Variable** | **Time 1 only** | | **Times 1 & 2** | | | $\boldsymbol{\chi}$**2/*t*-value** | |
|  | mean (SD) or % | n | | mean (SD) or % | n | |  |
| *Key demographics at T1* | | | | | | | |
| **Age** | 23.50 (7.45) | 445 | | 23.85 (8.15) | 190 | | *t* = 0.521 |
| **Gender** |  |  | |  |  | |  |
| Female | 65.5% | 291 | | 67.5% | 129 | | $\chi$^2^ = 0.457 |
| Male | 32.2% | 143 | | 30.9% | 59 | |  |
| Other | 2.3% | 10 | | 1.6% | 3 | |  |
| Total |  | 444 | |  | 191 | |  |
| **Ethnicity** |  |  | |  |  | |  |
| White/Caucasian | 53.1% | 237 | | 69.5% | 132 | | $\chi$^2^ = 15.624** |
| African/Caribbean/ Black British | 6.1% | 27 | | 3.2% | 6 | |  |
| Asian/Asian British | 30.5% | 136 | | 20% | 38 | |  |
| Mixed ethnicity | 6.7% | 30 | | 5.8% | 11 | |  |
| Other | 3.6% | 16 | | 1.6% | 3 | |  |
| Total |  |  | |  | 190 | |  |
| *Mental health outcome measures at T1* | | | | | | | |
| **GAD-7** | 8.89 (5.56) | 238 | | 8.70 (5.95) | 190 | | *t* = -0.342 |
| **PHQ-9** | 8.77 (5.42) | 238 | | 8.25 (5.76) | 190 | | *t* = -0.957 |
| **ISI-3** | 4.31 (3.72) | 238 | | 3.64 (3.45) | 190 | | *t* = -1.895 |
| **NIDA-ASSIST** | 2.62 (3.25) | 231 | | 2.19 (2.71) | 188 | | *t* = -1.429 |
| **SBQ-R** | 5.94 (3.26) | 233 | | 5.91 (2.90) | 189 | | *t* = -0.102 |

Notes. * *p* < 0.05, ** *p* <0.01, *** *p* < 0.001
